# Are We Neglecting Nontuberculous Mycobacteria Just as Laboratory Contaminants? Time to Reevaluate Things

**DOI:** 10.1155/2018/8907629

**Published:** 2018-06-21

**Authors:** Pooja Sharma, Digvijay Singh, Kusum Sharma, Santwana Verma, Sanjay Mahajan, Anil Kanga

**Affiliations:** ^1^Department of Microbiology, Dr Baba Saheb Ambedkar Medical College & Hospital, Rohini, New Delhi 110085, India; ^2^Department of Microbiology, Indira Gandhi Medical College, Shimla, Himachal Pradesh 171001, India; ^3^Department of Microbiology, PGIMER, Chandigarh 160012, India; ^4^Department of Microbiology, Dr YSPGMC, Nahan, Sirmaur, Himachal Pradesh 173001, India

## Abstract

**Objectives:**

Nontuberculous mycobacteria (NTM) incidences are on the rise worldwide, including the tuberculosis endemic areas. They should be identified rapidly to the species level and should be carefully differentiated as contamination, colonization, or disease. This study was aimed at determining the prevalence and clinicoepidemiological profile of mycobacteriosis cases.

**Materials and Methods:**

Cultures were made on liquid and solid media. NTM were identified by polymerase chain reaction (PCR) restriction analysis (PRA) and gene sequencing. Data was analyzed using Epi-info 7.

**Results:**

Out of the 1042 processed specimens, 16% were positive for M. tuberculosis complex and 1.2% for clinically significant NTM. M. intracellulare was the commonest species isolated. NTM were treated mainly on outdoor basis (92%), involving more extrapulmonary system (62%) and higher age-group of 41-60 years (69%). No significant factor was seen to be associated clinically, radiologically, and biochemically with the NTM infections.

**Conclusions:**

Our study highlights the importance of early diagnosis and differentiation among Mycobacterium tuberculosis and NTM so that these NTM are not underestimated in routine diagnostic procedures merely as environmental or laboratory contaminants.

## 1. Introduction

Mycobacteria other than tuberculosis (MOTT) also known as nontuberculous mycobacteria (NTM) and atypical mycobacteria are organisms that are normally found in environmental soil and water [1]. They have been recognized as a cause of human disease since 1950s. At that time, NTMs isolation had been rare, and they were found almost exclusively in patients with underlying diseases [2]. Recent reports have shown that mycobacteriosis is on the rise even in healthy people. The distribution of NTM is nonuniform which appears to be geographically or environmentally dependent but still remains poorly defined. Geographic differences in distribution of NTM have been investigated comprehensively by Hoefsloot et al. in Europe, America, Australia, and few parts of Asian continent [3].

Disease caused by NTM is nonnotifiable. NTM are important pathogens because of their high level of antitubercular drug (ATT) resistance [4, 5]. Recognizing the changing pattern of disease and epidemiology since 1980s with cases being reported in unrecognized populations, we initiated a study to assess the burden of such disease in our region [6, 7].

NTM are difficult to differentiate on the basis of clinical presentation from Mycobacterium tuberculosis complex (MTBC); thus it is necessary to identify NTM, as mere isolation especially from pulmonary sites does not establish the diagnosis. American Thoracic Society (ATS) guidelines are followed for the respiratory samples to correlate with clinical symptoms so that specific treatment can be instituted [5]. The aims of our study were (1) to determine isolation rate from pulmonary versus extrapulmonary sites, (2) to determine the patients' demographic characteristics and risk factors, (3) to determine effect of liquid versus solid culture on the isolation rate of NTM, and (4) to assess the disease incidence, the mycobacterial species involved, and the clinical conditions they caused.

## 2. Material and Methods

This prospective study was conducted in the tertiary care hospital at Shimla, Himachal Pradesh, India, over a period of one year from July 2013 to June 2014 on clinical samples from suspected patients of pulmonary and extrapulmonary mycobacterial infection.

### 2.1. Study Definitions

Differentiation between colonization and disease was done according to ATS 2007 guidelines in case of pulmonary specimens [5]. In extrapulmonary specimens, single isolation from sterile site as well as isolation for two or more times from nonsterile sites, along with the clinical correlation, was taken as confirmed disease [8].

### 2.2. Inclusion Criteria

Samples referred from patients admitted (IPD) or attending outdoor patient department (OPD) with clinical suspicion of pulmonary and extrapulmonary mycobacterial infection were included in the study.

### 2.3. Sample Collection and Transport

Sputum, bronchoalveolar lavage (BAL), body fluids, and aspirates (ascitic tap, pleural tap, synovial fluid, drains, pus discharge, gastric aspirates, and cerebrospinal fluid (CSF)) and early morning whole urine sample for three consecutive days collected in a sterile wide mouth screw capped container were received in the laboratory.

### 2.4. Sample Processing

Samples received were processed by digestion, decontamination, and concentration following the standard protocol [9]. Concentrated sample was divided into four parts.

- **First part: **This part was inoculated into MGIT 960 tube following manufacturer's instructions and tube was entered into the instrument [10]. From the positive tube, smear was prepared for Ziehl–Neelsen (ZN) and Gram's staining.
    If positive for acid-fast bacilli (AFB), it was reported as instrument-positive and AFB-positive. It was then screened for production of MPT 64 antigen using Immunochromatographic Assay (TBcID) [11].
    Those found positive for MPT 64 antigen were labeled as MTBC and those negative were labeled as NTM and were further identified by polymerase chain reaction (PCR) restriction analysis (PRA).
    The protocol for PRA was the one described by Theresa B, Taylor and coworkers [12]. Briefly, hsp65 region was amplified using Tb 11 and Tb 12 primers. Restriction digestion of hsp-65 PCR products was done with* Bst*EII and* Hae*III enzymes. PRA algorithm was used to identify the isolates. Representative specimens were further confirmed by sequencing at Post-Graduate Institute of Medical Education and Research, Chandigarh.
    If microorganisms other than acid-fast bacilli were present, they were reported as instrument-positive, AFB-negative, and contaminated.
    If no microorganisms were present on the smear, the tube was reentered into the instrument. Negative cultures were removed as “out of protocol” negatives after 42 days.
- **Second part: **This was inoculated on Lowenstein-Jensen (LJ) medium and incubated at 37°C. Bottles were examined daily for first 5–7 days and then weekly thereafter up to 8 weeks. If any growth was observed, a smear was made and heat fixed and ZN staining was done to confirm the AFB.
- **Third part: **It was inoculated into the blood agar plates to rule out any contamination.
- **Fourth part: **Smear was prepared, heat fixed, and stained by ZN method and examined under oil immersion.

### 2.5. Statistical Analysis

Data was entered on spreadsheet. Cross tabulation with outcome variable of interest was done using statistical software Epi-info version 7 (7.1.1.0).

## 3. Result

Out of the 1042 samples in the study, AFB were isolated from 18.6% (n=194) of samples. MTBC constituted 87% (168) and NTM were 13% (26) of total isolates.

Sensitivity of ZN, LJ, and MGIT-960 was 52%, 17%, and 97% for NTM.

NTM isolated from pulmonary sites were M.* intracellulare* 62.5% (5), M.* flavescens* 12.5% (1), M.* genavense* 12.5% (1), and M.* gordonae *12.5% (1).

Extrapulmonary NTM isolates included 6.5% (5) M.* intracellulare,* 2.6% (2) M.* abscessus,* 1.3% (1) M.* avium,* 1.3% (1) M.* mucogenicum,* 1.3% (1) M.* austroafricanum*, and 10.4%(8) M.* gordonae. *

Clinically significant NTM isolates were 50% (13) of the total NTM isolated in laboratory. They are summarized in Table 1. Among significant pulmonary isolates, 62.5%(5), i.e., 4 sputum and 1 BAL cases, fulfilled the criteria. In extrapulmonary cases, isolates from synovial fluid (1), pleural fluid (2), and urine (5) were clinically significant. All the patients were nonreactive for human immunodeficiency virus (HIV).

NTM were treated mainly on outdoor basis [92% (12)], involved extrapulmonary system more [62% (8)], and affected higher age-group [41-60 years (69%)]. History of tuberculosis was present in 31% (4) and radiological features of upper lung lobe involvement were seen in 60% of cases (Table 2).

NTM affected 41% (8) females and patients presented with fever and localized pain mainly [46% (6)]. 54% (7) of the patients were farmer by occupation. Erythrocyte sedimentation rate (ESR) was raised in 54% (7), all had normal total leukocyte count (TLC), and one patient (8%) had lymphocytosis.

## 4. Discussion

Mycobacteriosis being unnoticed in tuberculosis endemic areas is on the rise, being resistant to common antitubercular drugs [13]. Prevalence of clinically significant NTM among specimens received in our study was 1.2%. Burden of mycobacteriosis is still vague due to underdiagnosis and lack of awareness. Developing countries lack facilities and expertise and often overlook these infections at peripheral levels.

We observed that extrapulmonary system was more involved with respect to pulmonary system with NTM, but other researchers mainly found the higher involvement of pulmonary system [14–17]. Age-group of patients most commonly affected by NTM was 41-60 years (69%) in our study. Other authors found the mean age to range between 43 and 59 years [15–17]. It has been postulated that immunosenescence leads to weaker immune response in elderly individuals resulting in increased susceptibility to infections, including mycobacteriosis [18, 19].

NTM being opportunistic pathogens have less tendency to cause infection. In a study by Dirac et al., immunosuppression and prior lung disease were associated risk factors for mycobacteriosis [20]. But all the patients in our study were HIV nonreactive. Commonest risk factor found in pulmonary mycobacteriosis cases in our study was preexisting comorbid pulmonary conditions like past history of tuberculosis. Tobacco or alcohol use was not found to be statistically associated with mycobacteriosis. High risk of NTM infections among patients with prior history of ATT intake was reported in earlier studies which support our present observation [14, 16]. As stated earlier, mycobacteriosis among healthy individuals is not very rare [16]. Cases without recognized risk factors may have distinctive genetic susceptibility or environmental exposure to NTM [21]. Thus treating physician should keep mycobacteriosis in differential diagnosis in immunocompetent individuals also.

Pulmonary infections by NTM mostly involved the upper lobes of lungs as also observed by Costa et al. [22]. So it is difficult to differentiate this condition from tuberculosis which is also known to involve upper lobes more. Low hospitalization rate was seen among patients infected by NTM. This can be because of the mild pathogenic nature of NTM. Raised ESR though being very vague criterion in diagnosis of the NTM infection can be a contributory investigation. Neither increase nor decrease in blood cells count was seen with NTM infections. Thus, it is difficult to associate hematological laboratory parameters with NTM infection.

This study had few limitations. Firstly the number of patients with NTM isolates was small so as to conclude statistical differences. Secondly as this was tertiary care hospital based study, results might not represent the findings of population which could not attend this healthcare facility. Thus results may not be generalized to the community.

## 5. Conclusion

There is need for awareness regarding appropriate NTM diagnosis among physicians. NTM should be differentiated between colonization and disease. By using the rapid molecular methods, we can differentiate NTM from MTBC thus helping the clinician in early institution of specific treatment. PRA is cost-effective and can identify many mycobacterial species in a single experiment. These isolates are commonly disregarded as environmental contaminants in laboratories. One should be aware of increasing spectrum of these infections even in immunocompetent patients and more studies should be conducted to assess the importance and relevance of these isolates.

## Figures and Tables

**Table 1:** Clinical and microbiological features of clinically significant NTM.

Spm no.	Isolate	Spm	Chief complaints	H/O ATT	Age	Sex	No. of positive sputum samples
1.	M. intracellulare	Sputum	fever, productive cough	2	55	M	2

2.	M. intracellulare	Sputum	haemoptysis	2	22	F	2

3.	M. intracellulare	Sputum	haemoptysis	1	49	F	2

4.	M. intracellulare	Sputum	productive cough, SOB	3	40	M	3

5.	M. gordonae	BAL	cough	0	45	F	1

6.	M. intracellulare	Pleural Fluid	SOB	0	60	F	0

7.	M. intracellulare	Pleural Fluid	chest pain, anorexia	0	51	F	0

8.	M. intracellulare	Urine	haematuria, increased frequency	0	55	M	NA

9.	M. abscessus	Urine	sensation of incomplete evacuation	0	45	M	NA

10.	M. gordonae	Urine	right hypochondrium pain, vague lump in abdomen	0	54	F	NA

11.	M. intracellulare	Urine	increased frequency of urine	0	37	M	NA

12.	M. avium	Urine	pain right lumbar region	0	27	F	NA

13.	M. abscessus	Synovial Fluid	swelling right ankle	0	54	F	NA

NTM: nontuberculous mycobacteria; Spm: specimen; H/O ATT: history of antitubercular treatment; SOB: shortness of breath; NA: not applicable.

**Table 2:** Radiological features of patients with NTM isolates from sputum.

S. No	Isolate	Spm	Radiological features
1	M. intracellulare	Sputum	Patchy consolidation in right upper & bilateral lower lobes

2	M. intracellulare	Sputum	Cavitary lesion in right upper zone with in-homogeneous opacity in right middle zone

3	M. intracellulare	Sputum	Patchy consolidation in right upper lobe.

4	M. intracellulare	Sputum	Not available

5	M. gordonae	BAL	Not available

NTM: nontuberculous mycobacteria; Spm: specimen; BAL: bronchoalveolar lavage.

## Data Availability

The data used to support the findings of this study are available from the corresponding author upon request.
